# Self-Referred Walk-in (SRW) versus Emergency Medical Services Brought Covid-19 Patients

**DOI:** 10.30476/BEAT.2021.92229.1299

**Published:** 2022-01

**Authors:** Navid Kalani, Naser Hatami, Sajed Ali, Neema John Mehramiz, Fatemeh Rahmanian, Esmaeil Raeyat Doost, Marzieh Haghbeen, Samaneh Abiri, Mahdi Foroughian, Mohsen Ebrahimi

**Affiliations:** 1 *Research Center for Social Determinants of Health, Jahrom University of Medical Sciences, Jahrom, Iran*; 2 *Student Research Committee, Jahrom University of Medical Sciences, Jahrom, Iran*; 3 *Department of Biotechnology, University of Management and Technology, Sialkot, * *‎* *Pakistan* *‎*; 4 *Department of Psychiatry Neurology, Banner University Medical Center, Tucson, Arizona, USA*; 5 *Department of Emergency Medicine, Jahrom University of Medical sciences, Jahrom, Iran*; 6 *Women’s Health and Disease Research Center, Jahrom University of Medical Sciences, Jahrom, Iran*; 7 *Department of Emergency Medicine, Mashhad University of Medical sciences, Mashhad, Iran*

**Keywords:** Emergency medical services, Prehospital emergency care, COVID-19, Self-referral

## Abstract

**Objective:**

To compare the characteristics of the emergency medical services (EMS) brought COVID-19 patients versus self-referred walk-in patients.

**Methods:**

This was a Cross-sectional study of COVID-19 infected cases in Jahrom, south of Iran. Age, sex, the symptoms of beginning days’ passing, respiratory distress, PO2 at arrival, admission length and in-hospital death were retrieved for confirming COVID-19 cases in the whole 2020 year. Respiratory distress was considered as the sign that agitates the patient to call EMS care. Survival analysis was used to evaluate the possible difference of the hospitalization outcome in EMS brought or Self-referred walk-in (SRW) patients.

**Results:**

There was 704 (27.1%) registries patients transfer to the hospital by EMS and 1895 (72.9%) cases with SRW referred to the hospital. The survival distributions for the EMS group were statistically significant and lower than SRW group (*p*<0.05). Despite the SRW patients, respiratory distress was associated with lower survival in EMS group (*p*<0.05). Days passing the symptom’s beginning was significantly different between EMS group (6.1±5.3 days) and SRW group (6.9±4.6 days). Cox regression showed higher mortality rate in patients higher than 75 years old in both groups (*p*<0.05). Higher PO2 at arrival was associated with lower mortality rate of Hazard Ratio of 0.959 (*p*<0.001) and 0.903 (*p*<0.001) in EMS and SRW groups, respectively. The history of heart disease and hypertension were associated with 1.011 and 1.088 times more than mortality risk in EMS group; while cancer history was associated with 2.74 times more of mortality risk in SRW group.

**Conclusion:**

It seems that severe acute respiratory syndrome occurs soon in some patients that lead to the need for an ambulance to transfer the patient to the hospital. Therefore, EMS transfer patients should be considered for more risk of severe COVID-19; considering comorbidities of heart disease and hypertension as red flags.

## Introduction

Pre-hospital emergency is responsible for the free transfer of patients with various medical conditions. Ambulance service is one of the important and complementary components of pre-hospital emergency services (EMS). With the outbreak of coronavirus worldwide, EMS services have become an important part of the fight against COVID-19 and many EMS services are being assigned for COVID-19 suspected patients transfer [1]. In decision on dispatching an ambulance, transfer or non-transfer of emergency patients to hospitals are mostly based on the information exchange during telephone reports provided by patients or EMS technicians to physicians present at the message center. EMS and pre-hospital services play an important role to prevent and control of COVID-19 by having a high clinical suspicion of travel and contact history of febrile patients and patients with respiratory symptoms [2]. The technician clinical judgment or the telephone consulting physician opinion are important. Currently, the EMS services are being dispatched for patients with symptoms of fever, cough, dyspnea, intercostal or supraclavicular muscle retractions, stridor, and bloody sputum. Using of all health care facilities, capacities and synergy efforts are essential by given the timely identification importance of coronavirus suspected cases and health measures to limit the spread of the disease [3]. Suspected COVID-19 cases may call a pre-hospital emergency department or visit hospitals or community health centers. However, there is no complete information about the people’s behavior to choose these options. Various studies have shown that delayed symptom initial to hospitalization may be associated with worsen outcomes [4, 5]. Due to this issue, a detailed study of the patient’s characteristics that used pre-hospital services in compare with those who went directly to hospitals was included in the agenda of the present study. Our main objective was to compare the survival outcomes of COVID-19 patients based on their method of referral to medical center; while we should be considering the demographic information and the presence of the respiratory distress at arrival to the emergency department. 

## Materials and Methods

This study was a cross-sectional study of COVID-19 infected cases in Jahrom, south of Iran. The study was conducted based on the Guidelines and recommendations for ensuring Good Epidemiological Practice (GEP) [6]. Confirmation of Jahrom University of Medical Sciences (JUMS) research ethics committee was obtained (IR.JUMS.REC.1398.130). No identifiable information of any case was reported with respect to the patients’ autonomy. 

A check list of patient’s demographic and clinical data was available at emergency department which filled by nurses on patient’s admission time. Fulfilled datasets were acquired from Governmental agencies of Jahrom University of Medical Sciences in whole 2020. 

The study was done at Motahari and Peymanieh Hospitals of Jahrom city in the whole year of 2020. We included all samples through the 2020 year (Census sampling); while excluding the cases with missing information

The endpoints of study include age, sex, days passing the symptom’s beginning, have respiratory distress, PO2 at arrival, admission length and in-hospital death. Patient higher than 18 years old were studied. Manifestations of deep breathing and/or respiratory rate of higher than 20 per minute (tachypnea) was considered respiratory distress which was reported by EMS technician or COVID-19 emergency department triage personnel. Time of hospitalization to death was considered as a study event and hospitalization time to discharge was as censoring for survival analysis. Respiratory distress was considered the sign that agitate patient to call EMS care based on a pilot interview with Jahrom city EMS care telephone center. COVID-19 associated death that happened at hospital had to be confirmed by infectious disease specialist and patient’s cadaver had to be buried based on COVID-19 protocols. 

Total number of 3832 patients were registered for the study. Registers with unknown and missed variables of interest were excluded. Registers were filtered for patient’s recruitment with a stratification of EMS brought or SRW. In this study, 704 entries were recruited in EMS brought group and 1895 in SRW group based on this stratification. The data were first descriptively analyzed and the results were presented as n (%) for categorical variables and mean ± SD for continuous variables. Right-tailed Goodness of fit test was assessed that had more than 2 levels in case of age categories; while observed and expected frequencies were not close. The median survival of patients with COVID-19 was compared with each other by referral to hospital condition, age groups, sex, and respiratory distress at arrival, using the Kaplan-Meier method. Multivariable cox proportional hazards regression model was used for each group of EMS and SRW patients based on the variables with significant results of Kaplan-Meier method. SPSS software version 24 was used. P-value lower than 0.05 was considered statistically significant. 

## Results

There were 704 (27.1%) registries of patients brought to hospital by EMS and 1895 (72.9%) cases with SRW referral to hospital. Mean age of patients in EMS group was 62.1±19.2 years and 53.4±18.4 years old in SRW group with a statistically significant difference (*p*<0.001). There were no differences in term of sex distribution (*p*=0.869). Days passing the symptom’s beginning was significantly different between EMS group (6.1±5.3 days) and SRW group (6.9±4.6 days) (*p*=0.011). PO2 at arrival of EMS group was significantly lower than SRW group (*p*<0.001). But there was no significant difference at arrival (*p*=0.104) and hospitalization length (*p*=0.139) in term of having Respiratory distress as shown in Table 1. 

**Table 1:** Basal characteristics of registries

	EMS N=704		SRW N=1895		p value
	Mean or Median /n	SD/%	Mean or Median/n	SD or IQR/%	
Age, years, mean	62.1	19.2	53.4	18.4	<0.001a
Sex, male, n	380	54	1016	53.6	0.869b
Time passing the symptom’s beginning, days, mean	6.119	5.3	6.97	4.7	0.011c
Having Respiratory distress, mean	453	64.3	1104	58.2	0.104b
Hospitalization length, days, mean	4.22	4.2	3.9	3.9	0.139a
PO2 at arrival, %, mean	88.2	9.4	90.7	7.6	<0.001a

^a^ Independent T-test; b Fisher exact test; c Mann–Whitney U test

**Fig. 1:**
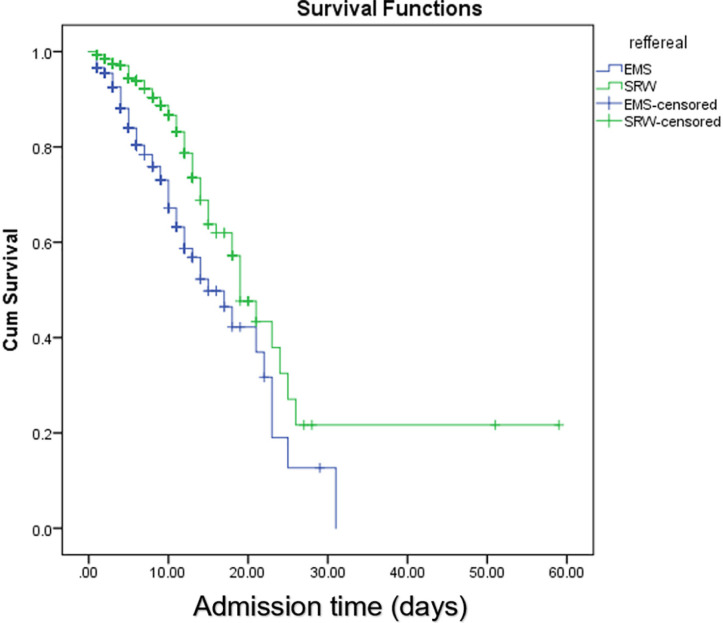
Kaplan Meier curves of survival in emergency medical services versus self-referred walk-in group

A log rank test was run to determine the differences in the survival distribution for the different types of referrals to emergency department. The survival distributions for the EMS or SRW were statistically significant different (*p*<0.001), (Figure 1). 

In the present study, 101 cases died in EMS group (14.7%) and 96 in SRW group (0.5%). Based on the Log Rank test, the survival distributions for age groups of under 25, 25 to 49, 50 to 75, and more than 75 years old were statistically significantly different, in both EMS (*p*<0.001) and SRW (*p*<0.001) groups. The survival distributions for the sex were not statistically significant different in both EMS (*p*=0.903) and SRW (*p*=0.112). The survival distributions for having respiratory distress were statistically significant different in EMS (*p*=0.009) group; but it was not significant in SRW (*p*=0.611) group (Table 2).

**Table 2:** Log Rank test of the survival distributions.^a^

		**EMS**					**SRW**				
		***p***	**Median**	**SE**	**95% Confidence Interval**		***p***	**Median**	**SE**	**95% Confidence Interval**	
					**Lower Bound**	**Upper Bound**				**Lower Bound**	**Upper Bound**
Gender	Men	0.903	17.0	3.5	10.1	23.9	0.112	18.0	2.6	12.7	23.2
	Women		14.0	2.3	9.4	18.5		19.0	2.1	14.8	23.1
Respiratory distress		0.009	15	4	7.1	22.8	0.611	19	3.1	12.9	25.1

^a^ Median survival of age groups was not calculated as the estimated survival probability reached 50% in some subgroups.

Cox regression showed that age group of under 25 years have hazard ratio (HR) of 0.435 for mortality in EMS group but age under 25 years and 25-49 years had HR of 0.284 and 0.026 mortality in SRW group, respectively, in comparison with reference age group (higher than 75 years old). Sex was not associated with different risk of mortality. Higher PO2 at arrival was associated with lower mortality rate of HR 0.9 (*p*<0.001) and 0.9 (*p*<0.001) in EMS and SRW groups, respectively. Respiratory distress was associated with 2.36 times of mortality risk in EMS and 2.3 times in SRW group. Among the comorbidities, heart disease history and hypertension were associated with 1.011 and 1.088 times of mortality risk in EMS group; while cancer history was associated with 2.74 times in SRW group (Table 3). 

**Table 3:** Cox proportional hazards regression

		**EMS**				**SRW**			
		***p***	**HR**	**95.0% CI**		***p***	**HR**	**95.0% CI**	
				**Lower**	**Upper**			**Lower**	**Upper**
Age	>75, reference	0.054	-	-	-	0.000	-	-	-
	50-75	0.978	-	-	-	0.970	-	-	**-**
	25-49	0.093	0.3	0.1	1.1	0.000	0.0	0.0	0.1
	<25	0.011	0.4	0.2	0.8	0.000	0.2	0.1	0.5
Days passing the symptom’s beginning		0.563	1.0	0.9	1.1	0.228	0.9	0.9	1.0
Female Sex		0.931	1.0	0.6	1.5	0.118	0.7	0.5	1.1
Po2 at arrival		<0.001	0.9	0.8	1	<0.001	0.9	0.8	1
Respiratory distress		<0.001	2.3	1.4	3.7	<0.001	2.3	1.5	3.6
HRCT with positive findings		0.061	-	-	-	0.479	-	-	-
Cancer		0.260	1.6	0.6	3.9	0.041	2.7	1.0	7.2
Liver disease		-	-	-	-	-	-	-	-
Diabetes mellitus		0.075	1.4	0.9	2.2	0.296	1.3	0.8	2.1
Hematologic disease		0.528	1.4	0.4	4.8	0.960	1.1	0.1	7.9
Pregnancy		-	-	-	-	-	-	-	-
Heart disease history		0.045	1.6	1.0	2.4	-	-	-	-
Kidney Disease		0.172	1.6	0.8	3.1	-	-	-	-
Asthma		0.866	0.9	0.3	2.5	0.242	1.4	0.8	2.3
Respiratory disease		0.636	1.3	0.4	4.3	0.693	1.2	0.4	3.4
Neurologic disease		0.760	1.2	0.4	2.9	0.314	1.6	0.7	3.7
Hypertension		0.019	1.7	1.1	2.5	0.294	2.2	0.5	9.7

## Discussion

The present study showed that some individuals appear to develop severe acute respiratory syndrome earlier than the majority of patients who need emergency transfer to the hospital. The study showed that most COVID-19 cases (72.92%) refer to hospital theirselves. In the cases which are brought by EMS, may have worse prognosis than SRW patients. As Satty *et al*., [1] stated, respiratory calls for EMS had increased in comparison of pre-COVID-19 years. While we did not compare our dataset with previous years; increased respiratory calls due to COVID-19 in pandemic era may need more attention as we find a worse prognosis in EMS encounter patients [1]. Days passing the symptom’s beginning was significantly different between EMS group and SRW group. Interpretation of this finding can be inconsistent with the public’s behavioral response to the disease or the course of the disease. In the social view, we expected patients who referred on their own to have shorter onset of symptoms. But the results showed that patients brought in by the EMS had less time passing the symptoms’ onset. This could reject that patient may not refer to medical care due to various social aspects, like being not familiar to COVID-19 symptoms or social stigma. But our study was not in a position to address this issue; while mean 6 days passing the symptom onset is yet too long and needs more evaluation. 

It seems that patients with severe symptoms like self-reported dyspnea and respiratory distress were hospitalized and evaluated in the present study based on hospitalization criteria which is being used in our health care system for COVID-19 patients. The main indication for hospitalization of COVID-19 patients in our medical centers was the presence of respiratory distress or oxygen saturation level less than 93% in the room air or respiratory rate of higher than 30 (with or without fever), in addition to the physician clinical judgment. Various studies [7, 8] have shown that occurrence of respiratory distress was reported to be 5 to 8 days after the symptom’s onset. Our study showed that this condition could have a wider range and will happen very soon in some patients. Consequently, this may lead to a respiratory call for EMS; while having respiratory distress was not associated with time passing of symptoms in EMS group patients. It is happening in longer time passing the symptoms onset in SWR patients. 

On the other hand, no previous study has evaluated these differences among EMS brought versus SRW patients. Therefore, there may be an indispensable need to assess the root factors beyond the findings of this study as well as social and public issues or biological aspects. 

Main finding of our study had lower survival rate of the EMS group in compare with SRW group. Respiratory distress at arrival and hospitalization length were not significantly different among both groups. 

Heart disease history and hypertension were associated with 1.011 and 1.088 times more risk of mortality in EMS group. While we find a small increased probability of mortality in patients who has heart disease history or hypertension; many studies have confirmed higher risk of mortality among them [9, 10]. 

In conclusion, some patients seem to have serious acute respiratory syndrome earlier than most other patients that necessitate using of an ambulance to transport them to the hospital. As a result, EMS-transported patients should be assessed for a higher risk of severe COVID-19 as our study showed higher rate of death in compare with SRW patients. Also, comorbidities are representing as red flags such as heart disease and hypertension. ‎

*Study Strength and Limitations*

To our knowledge, this was the first study evaluating patients based on the referral method to medical center and our study had a high population size; while there are some limitations to be addressed. First of all, more detailed illness history is needed to detect variables affecting symptom onset to referral time. As this study was conducted in one year, the definitively public perceptions and knowledge might have been received through one year passing the pandemic and there may be some differences with current results if it will be compared in various timeframes. Therefore, the public response evaluation to disease symptoms may be needed to be compared in different epidemic peaks. Based on our pilot findings, we hypothesized that respiratory distress is responsible for the EMS calls in most cases. But many other factors are affecting the public behavior in using of pre-hospital cares. Also, the COVID-19 treatment protocol has been changed multiple times that could potentially has biased our results. Despite the high sample size, there should be caution in our results of generalizability and clinical approach as more detailed assessment of other clinical and social factors. 

## Declarations

### Ethics approval and consent to participate

The institutional review board (IRB) of Jahrom University of Medical Sciences (JUMS) approved this study with Code of IR.JUMS.REC.1398.130. Respecting the patients’ autonomy, no identifiable information was reported. 

### Consent for publication

Not Applicable. 

### Conflict of interests

There are no conflicts of interest in this study.

### Funding support

None. 

### Authors' contributions

All the authors met the criteria of authorship based on the recommendations of the international Committee of Medical Journal Editors.

### Acknowledgements

We would like to thank the Clinical Research Development Unit of Peymanieh Educational and Research and Therapeutic Center of Jahrom University of Medical Sciences for providing facilities to this work.
